# Early-Stage Chronic Kidney Disease and Related Health Care Spending

**DOI:** 10.1001/jamanetworkopen.2023.51518

**Published:** 2024-01-12

**Authors:** Naomi Sakoi, Yuichiro Mori, Yusuke Tsugawa, Junko Tanaka, Shingo Fukuma

**Affiliations:** 1 Department of Epidemiology, Infectious Disease Control and Prevention, Graduate School of Biomedical and Health Sciences, Hiroshima University, Hiroshima, Japan; 2 Human Health Sciences, Kyoto University Graduate School of Medicine, Kyoto, Japan; 3 Division of General Internal Medicine and Health Services Research, David Geffen School of Medicine at UCLA, Los Angeles, California; 4 Department of Health Policy and Management, UCLA Fielding School of Public Health, Los Angeles, California

## Abstract

**Importance:**

The global burden of chronic kidney disease (CKD) is substantial and potentially leads to higher health care resource use.

**Objective:**

To examine the association between early-stage CKD and health care spending and its changes over time in the general population.

**Design, Setting, and Participants:**

Cohort study using nationwide health checkup and medical claims data in Japan. Participants included individuals aged 30 to 70 years with estimated glomerular filtration rates (eGFR) of 30 mL/min/1.73 m^2^ or greater at the baseline screening in 2014. Data analyses were conducted from April 2021 to October 2023.

**Exposure:**

The CKD stages at baseline, defined by the eGFR and proteinuria, were as follows: eGFR of 60 mL/min/1.73 m^2^ or greater without proteinuria, eGFR of 60 mL/min/1.73 m^2^ or greater with proteinuria, eGFR of 30 to 59 mL/min/1.73 m^2^ without proteinuria, and eGFR of 30 to 59 mL/min/1.73 m^2^ with proteinuria.

**Main Outcome and Measures:**

The primary outcome was excess health care spending, defined as the absolute difference in health care spending according to the baseline CKD stages (reference group: eGFR ≥60 mL/min/1.73 m^2^ without proteinuria) in the baseline year (2014) and in the following 5 years (2015 to 2019).

**Results:**

Of the 79 988 participants who underwent a health checkup (mean [SD] age, 47.0 [9.4] years; 22 027 [27.5%] female), 2899 (3.6%) had an eGFR of 60 mL/min/1.73 m^2^ or greater with proteinuria, 1116 (1.4%) had an eGFR of 30 to 59 mL/min/1.73 m^2^ without proteinuria, and 253 (0.3%) had an eGFR of 30 to 59 mL/min/1.73 m^2^ with proteinuria. At baseline, the presence of proteinuria and an eGFR less than 60 mL/min/1.73 m^2^ were associated with greater excess health care spending (adjusted difference, $178; 99% CI, $6-$350 for proteinuria; $608; 99% CI, $233-$983 for an eGFR of 30-59 mL/min/1.73 m^2^; and $1254; 99% CI, $134-$2373 for their combination). The study consistently found excess health care spending over the following 5 examined years.

**Conclusions and Relevance:**

In this cohort study of nationwide health checkup and medical claims data in Japan, early-stage CKD was associated with excess health care spending over the 5 examined years, and the association was more pronounced with a more advanced disease stage.

## Key Points

**Question:**

What is the association between early-stage chronic kidney disease (CKD) and health care spending and its changes over time in the general population?

**Findings:**

In this cohort study of 79 988 participants, early-stage CKD was associated with greater excess health care spending over 5 years. Mildly reduced estimated glomerular filtration rates, proteinuria, and their combination were independently associated with excess health care spending even after adjusting for hypertension and diabetes.

**Meaning:**

The findings of this study suggest the importance of designing actions to prevent the progression of CKD and reduce the health care burden associated with CKD for a sustainable health care system.

## Teaser

This cohort study evaluates the association between early-stage chronic kidney disease and health care spending and its changes over time in Japan.

## Introduction

Chronic kidney disease (CKD) has a global prevalence of 9.1%^1^ and is a significant risk factor for end-stage kidney disease (ESKD)^2^ and cardiovascular disease (CVD).^3,4^ When CKD progresses to ESKD or triggers CVD, significant utilization of health care resources is inevitable.^5,6,7,8^ Furthermore, there is a substantial number of patients with early-stage CKD in the general population^9,10,11,12^ who are unaware of their CKD status. CKD has the potential to increase health care spending through utilization of health care services and the spending incurred by the treatment of CKD complications. However, despite the central importance of CKD as potentially associated with health care spending growth, evidence is limited regarding the economic burden of early-stage CKD for the population.

Existing studies on this topic have found that advanced-stage CKD (mainly in patients who received diagnoses from physicians) was associated with higher health care utilization. However, there were several limitations to these studies, hindering efforts to develop interventions to effectively control excess health care spending associated with CKD. For example, previous studies^7,8,13,14^ analyzing the data of patients who received CKD diagnoses using hospital or registry data might have underestimated the economic burden of early-stage CKD. Claims data used for health services research in many countries often lack information on laboratory tests (urine test and serum creatinine test), precluding the investigators studying the impact of CKD using those data. In Japan, we have a unique opportunity to enable the examination of early CKD stages in the general population based on a nationwide annual health checkup program.^15^ By examining the economic burden of early-stage CKD, physicians, patients, policy makers, and public health sectors can consider prioritizing interventions for this population.

In this context, our objective was to examine excess health care utilization (specifically, health care spending, days of outpatient treatment, and hospitalizations) according to proteinuria and the estimated glomerular filtration rate (eGFR) among individuals with early-stage CKD in the general population using nationwide health checkup data in Japan.

## Methods

### Data Source and Study Design

We analyzed national annually collected health checkup and medical claims data collected between January 1, 2014, and December 31, 2019, from 1 of the largest employment-based health insurers in Japan: the National Health Insurance Association for Civil Engineering and Construction. The data included basic demographic information (ie, age, sex, and so forth), medical claims data for outpatient and inpatient care, and annual health checkup data (eGFR, urine protein levels detected using dipstick tests, blood pressure values, hemoglobin A_1c_ [HbA_1c_] levels, low-density lipoprotein cholesterol levels, and self-reported medication use [antihypertensive, antidiabetic, and antihyperlipidemic drugs]). Individuals with a diastolic blood pressure of 90 mm Hg and higher or a systolic blood pressure 140 mm Hg and higher or those using hypertension medication were classified as having hypertension. Moreover, those with an HbA_1c_ level 6.5% and higher (to convert to proportion of total hemoglobin, multiply by 0.01) or using medication for diabetes were classified as having diabetes. For the cross-sectional design, we used data from the baseline year (2014) both for the CKD stages and health care utilization. For the cohort design, we used baseline year data (2014) for the CKD stages, and the following years’ data (from 2015 to 2019) for the health care utilization. This study adhered to the Strengthening the Reporting of Observational Studies in Epidemiology (STROBE) reporting guideline for cohort studies. This study was conducted in accordance with the principles of the Declaration of Helsinki. The institutional review board of Kyoto University approved this study. As we only analyzed anonymized data from the database, the need for informed consent was waived.

### Annual Health Checkup Program

In Japan, all employers should offer an annual health checkup program for their employees, regardless of the employees’ health status. Approximately half of individuals undergo additional eGFR tests, which may be added on a company-by-company basis (and not at the discretion of the individual). If CKD is detected during the health checkup, the patient is informed of the results and recommended to visit a physician. Given that the health checkups are conducted annually regardless of health status and could be linked with longitudinal medical claims data, we had a unique opportunity to capture all CKD stages and examine both the cross-sectional and longitudinal associations with health care utilization.

### Participants

We analyzed data of individuals aged 30 to 70 years with an eGFR of 30 mL/min/1.73 m^2^ or higher in 2014 (year 0) who had not been treated with dialysis therapy. Participants were followed up until 2019 (year 5) or loss of eligibility for health insurance.

### Exposures

The eGFR was calculated from the serum creatinine test values using a standard formula.^16,17^ According to the Kidney Disease Improving Global Outcomes (KDIGO) classification in 2012,^18^ we defined baseline CKD stages using the results from 2014. Regarding the classification of CKD stages, since the number of participants in stage G3b (eGFR 30-44 mL/min/1.73m^2^) was limited, stages G3b and G3a (eGFR 45-59 mL/min/1.73m^2^) were combined into 1 category. For proteinuria, this study defined stage A2-3 as a result of 1 or higher on a dip-stick urine test. Finally, patients were classified into 4 groups based on 2 binary categories: mildly reduced eGFR (G3 [eGFR 30-59 mL/min/1.73 m^2^]) and proteinuria (A2-3 [1 or higher]). The groups were as follows: reference without CKD (G1-2 and A1), only proteinuria (G1-2 and A2-3), only mildly reduced eGFR (G3 and A1), and both mildly reduced eGFR and proteinuria (G3 and A2-3).

### Outcomes

The primary outcome was excess health care spending, which was defined as the absolute difference in US dollars for individuals across different CKD stages compared with those without CKD at baseline. The conversion from Japanese yen to US dollars was based on the exchange rate as of November 11, 2023 ($1 = ¥149.03). In Japan, under a universal health insurance system, which is financed mostly by taxes, users pay 10% to 30% of the medical costs (depending on their age, income, and family structure), and the insurer pays the rest.^19^ In this analysis, we defined health care spending as the total amount of money for health care utilization paid by both the user and the insurer for any medical care. The secondary outcomes included the absolute difference in the number of outpatient care visits and hospitalization days, assessed separately. Excess health care utilization was analyzed cross-sectionally in 2014 (year 0) and longitudinally in 2015 (year 1), 2017 (year 3), and 2019 (year 5).

### Statistical Analysis

We examined excess health care spending in individuals in each stage of CKD, adjusting for age, sex, diabetes, and hypertension.^20^ Excess health care spending, along with excess days of outpatient care and hospitalization, included absolute differences compared with a reference group. We applied a 2-part model due to the typically high prevalence of 0 expenditures and the skewed distribution of the data.^21,22,23^ In the first part, a probit model was used to estimate the probability of any health care spending. In the second part, a generalized linear model with a gamma distribution and log link was used to estimate the spending amount of people who incurred expenses. These analyses were both cross-sectional (CKD stage and outcomes measured concurrently at baseline) and longitudinal (CKD stage at baseline with outcomes tracked in subsequent years). To address the issue of multiple comparisons in our primary analysis, we present 99% CIs, rather than the standard 95% CIs. We considered a difference between groups to be statistically significant when the confidence interval did not include 0. Statistical analyses were carried out using R version 4.1. (R Project for Statistical Computing) and Stata version 17.1 (StataCorp). Data were analyzed from April 2021 to October 2023.

For the secondary analysis, first, we estimated excess health care spending with adjustment only for age and sex (eFigure 1 in Supplement 1). Second, subgroup analyses were performed based on age (≥60 or <60 years) (eFigure 2 in Supplement 1), sex (female or male) (eFigure 3 in Supplement 1), diabetes (eFigure 4 in Supplement 1), and hypertension (eFigure 5 in Supplement 1). Third, to consider the frequency of CKD progression during the follow-up, we calculated the proportion of participants who progressed to an eGFR less than 30 mL/min/1.73 m^2^ or started dialysis therapy over the 5 examined years by the baseline CKD stages (eFigure 6 in Supplement 1). Fourth, to examine the validity of the CKD staging, we reanalyzed the data using a more detailed CKD staging according to KDIGO guidelines^18^ (eFigure 7, eFigure 8, and eFigure 9 in Supplement 1): G1 and A1 (reference), G1 and A2-3, G2 and A1, G2 and A2-3, G3a and A1, G3a and A2, G3a and A3, G3b and A1, and G3b and A2-3. Fifth, to explore practice details specific to CKD, we calculated the excess use of antihypertensive and antidiabetic drugs (eFigure 10 in Supplement 1). Sixth, to accommodate the skewed distribution of health care spending, we have presented the mean values of health care spending for each decile, as well as the median and IQR, across different CKD stages (eFigure 11 in Supplement 1).

## Results

### Participant Characteristics

Of the 79 988 participants who underwent a health checkup (mean [SD] age, 47.0 [9.4] years; 22 027 [27.5%] female), 2899 (3.6%) had an eGFR of 60 mL/min/1.73 m^2^ or greater with proteinuria, 1116 (1.4%) had an eGFR of 30 to 59 mL/min/1.73 m^2^ without proteinuria, and 253 (0.3%) had an eGFR of 30 to 59 mL/min/1.73 m^2^ with proteinuria (Table). In summary, 4268 participants (5.3%) had early-stage CKD (eGFR 30 to 59 mL/min/1.73 m^2^ or proteinuria). Participants with an eGFR of 30 to 59 mL/min/1.73 m^2^ with proteinuria were older, more likely to be male, and more likely to have hypertension and diabetes. Among the 79 988 participants, 75 537 (94.4%), 68 587 (85.7%), and 63 425 (79.3%) were followed up until 2015 (year 1), 2017 (year 3), and 2019 (year 5), respectively.

**Table.** Participant Characteristics According to Chronic Kidney Disease Stages at Baseline

Characteristic	Participants, No. (%)				
	Overall (N = 79 988)	eGFR ≥60 mL/min/1.73 m^2^		eGFR 30-59 mL/min/1.73 m^2^	
		Without proteinuria (n = 75 720)	With proteinuria (n = 2899)	Without proteinuria (n = 1116)	With proteinuria (n = 253)
Age, mean (SD), y	47.0 (9.4)	46.8 (9.3)	47.5 (9.5)	58.6 (7.2)	56.3 (8.8)
Sex					
Female	22 027 (27.5)	21 391 (28.3)	519 (17.9)	98 (8.8)	19 (7.5)
Male	57 961 (72.5)	54 329 (71.7)	2370 (82.1)	1018 (91.2)	234 (92.5)
Blood pressure, mean (SD), mm Hg					
Systolic	123.4 (16.6)	123.1 (16.3)	129.6 (20.0)	130.5 (18.3)	136.0 (20.6)
Diastolic	75.9 (12.0)	75.6 (11.9)	80.6 (13.6)	81.1 (12.2)	82.5 (13.3)
Hemoglobin A_1c_, mean (SD), %	6.1 (7.4)	6.1 (7.4)	6.3 (5.7)	6.3 (6.9)	7.7 (11.8)
LDL cholesterol, mean (SD), mg/dL	128.4 (45.9)	128.3 (45.7)	132.3 (55.1)	129.7 (34.1)	126.0 (35.1)
Medication use					
Antihypertensive drugs	9484 (11.9)	8118 (10.7)	669 (23.1)	526 (47.1)	171 (67.6)
Antidiabetic drugs	2856 (3.6)	2321 (3.1)	330 (11.4)	137 (12.3)	68 (26.9)
Comorbidities^a^					
Hypertension	20 025 (25.0)	17 851 (23.6)	1272 (43.9)	702 (62.9)	200 (79.1)
Diabetes	5345 (6.7)	4480 (5.9)	590 (20.4)	188 (16.8)	87 (34.4)
eGFR, mean (SD), mL/min/1.73 m^2^	85.1 (9.7)	85.8 (8.7)	84.0 (9.7)	54.0 (5.8)	48.8 (7.8)

Abbreviations: eGFR, glomerular filtration rate; LDL, low-density lipoprotein.

To convert LDL cholesterol to millimoles per liter, multiply by 0.259; hemoglobin A_1c_ levels to proportion of total hemoglobin, multiply by 0.01.

^a^ Participants who have comorbidities based on the results of the health checkup. Hypertension: systolic blood pressure greater than 140 mm Hg, diastolic blood pressure greater than 100 mm Hg, or on medication for hypertension; Diabetes: hemoglobin A_1c_ level greater than 6.5% or receiving medication for diabetes.

### Excess Health Care Spending

**Figure 1.**
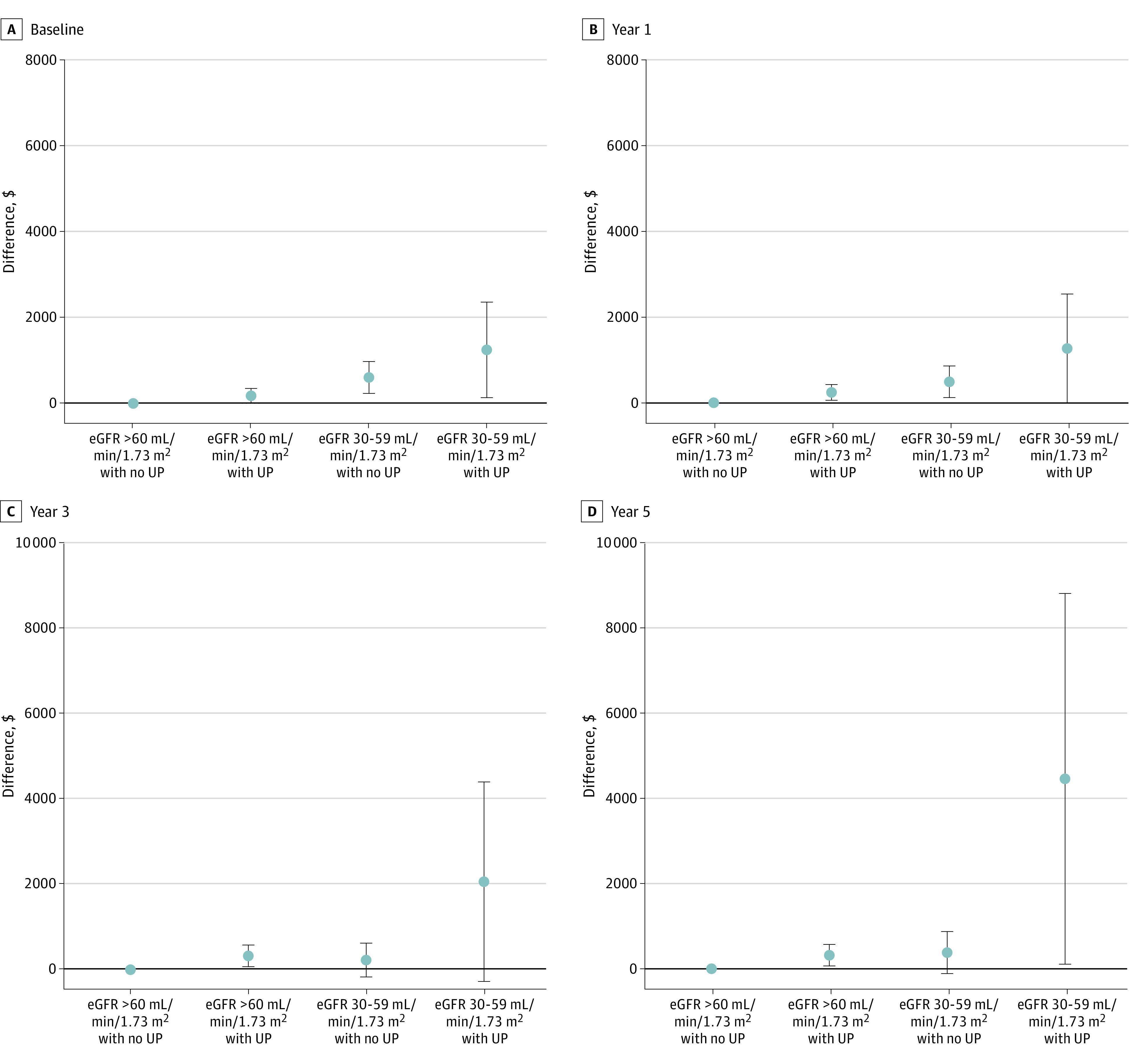
Excess Health Care Spending for Each Chronic Kidney Disease Stage Baseline year was 2014; year 1: 2015; year 3: 2017; year 5: 2019. Excess health care spending is expressed as the difference in health care spending (US dollars) for each stage of chronic kidney disease compared with the reference group (estimated glomerular filtration rate [eGFR] ≥60 mL/min/1.73 m^2^ and nonproteinuria), adjusting for age, sex, hypertension, and diabetes. Error bars show 99% CIs. UP indicates urinary protein.

In a cross-sectional analysis at baseline, we found significant excess health care spending to be associated with proteinuria and mildly reduced eGFR (adjusted difference, $178; 99% CI, $6-$350 for proteinuria; $608; 99% CI, $233-$983 for an eGFR of 30 to 59 mL/min/1.73 m^2^; and $1254; 99% CI, $134-$2373 for their combination) (Figure 1). Longitudinally over the 5 examined years, we found consistent excess health care spending associated with proteinuria and mildly reduced eGFR. For participants with a combination of proteinuria and mildly reduced eGFR, we found a longitudinal increase in excess health care spending over the 5 examined years.

### Excess Days of Outpatient Care and Excess Hospitalizations

**Figure 2.**
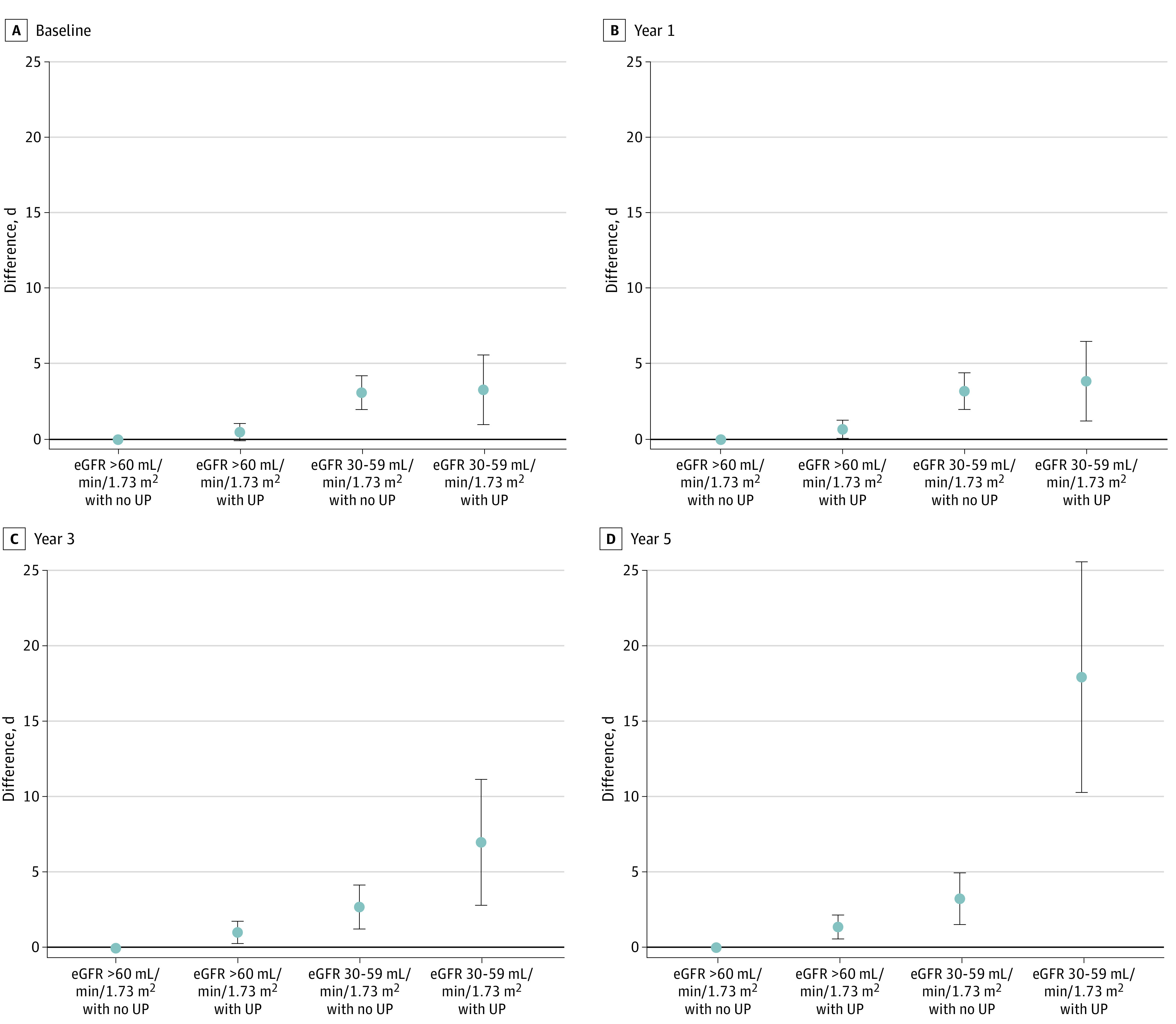
Excess Days of Outpatient Care for Each Chronic Kidney Disease Stage Baseline year was 2014; year 1: 2015; year 3: 2017; year 5: 2019. Excess days of outpatient care is expressed as the difference in the number of days of outpatient care for each stage of CKD compared with the reference group (estimated glomerular filtration rate [eGFR] ≥60 mL/min/1.73 m^2^ and nonproteinuria), adjusting for age, sex, hypertension, and diabetes. Error bars show 99% CIs. UP indicates urinary protein.

**Figure 3.**
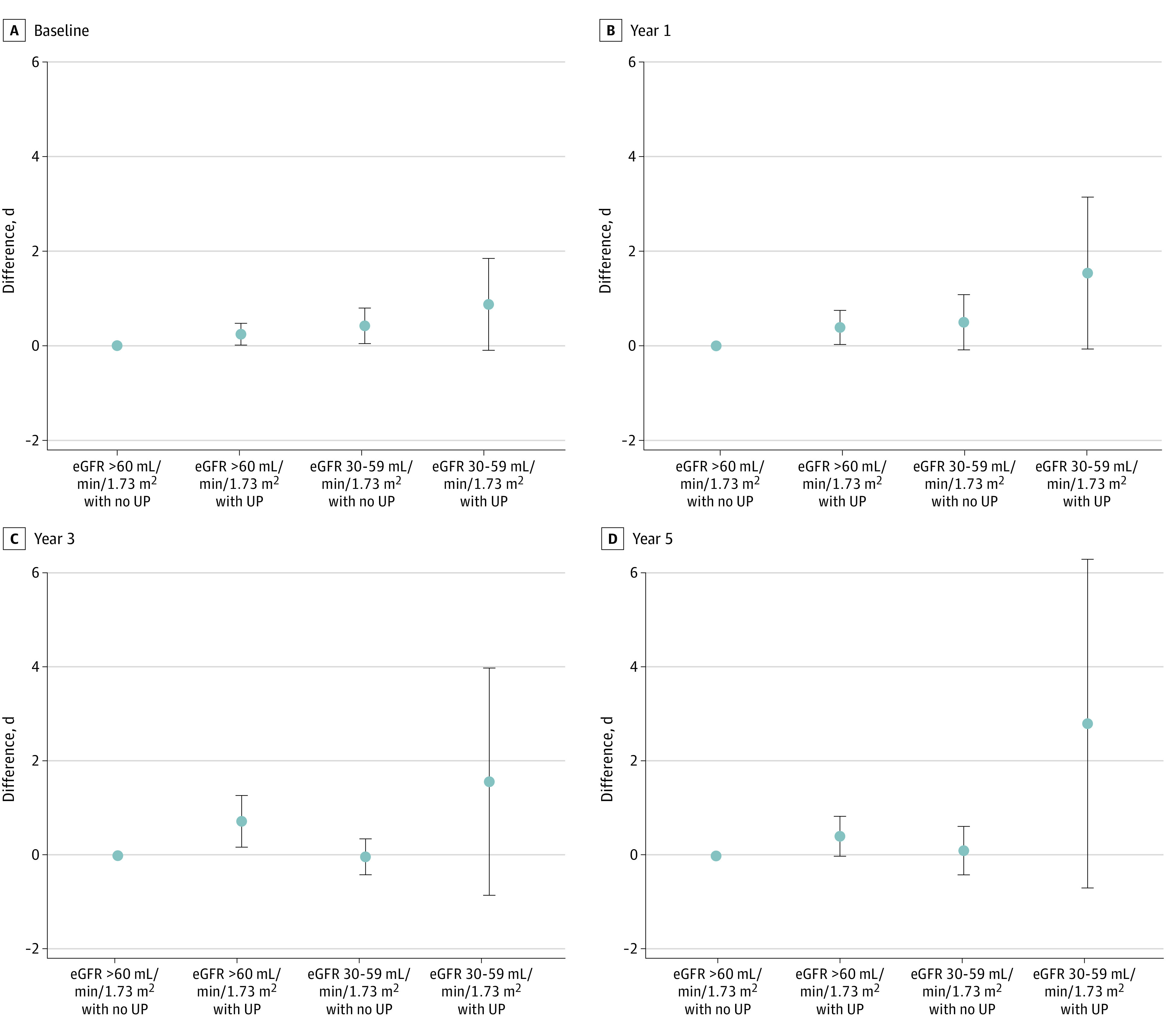
Excess Days of Hospitalization for Each Chronic Kidney Disease Stage Baseline year was 2014; year 1: 2015; year 3: 2017; year 5: 2019. Excess days of hospitalization is expressed as the difference in the number of days of inpatient care for each stage of CKD compared with the reference group (estimated glomerular filtration rate [eGFR] ≥60 mL/min/1.73 m^2^ and nonproteinuria), adjusting for age, sex, hypertension, and diabetes. Error bars show 99% CIs. UP indicates urinary protein.

In a cross-sectional analysis at baseline, we found significant excess days of outpatient care to be associated with mildly reduced eGFR (adjusted difference, 3.11 days; 99% CI, 1.99 to 4.23 days for an eGFR of 30 to 59 mL/min/1.73 m^2^ without proteinuria; and 3.30 days; 99% CI, 0.99 to 5.60 days for an eGFR of 30 to 59 mL/min/1.73 m^2^ with proteinuria) (Figure 2). In a cross-sectional analysis at baseline, we found significant excess days of hospitalization to be associated with proteinuria and mildly reduced eGFR (adjusted difference, 0.24 days; 99% CI, 0.01 to 0.47 days for proteinuria; 0.42 days; 99% CI, 0.04 to 0.80 days for an eGFR of 30 to 59 mL/min/1.73 m^2^; and 0.87 days; 99% CI, −0.10 to 1.85 days for their combination) (Figure 3). The excess days of hospitalization associated with proteinuria were consistently observed over the 5 examined years.

### Secondary Analysis

When we did not adjust for hypertension and diabetes, we found greater excess health care spending than when we did adjust for these variables (eFigure 1 in Supplement 1). According to the subgroup analysis by age, excess health care spending was greater in the younger subgroup (<60 years) than in the older subgroup (≥60 years) (eFigure 2 in Supplement 1). Excess health care utilization was similar between female and male individuals (eFigure 3 in Supplement 1). Excess health care spending was not greatly associated with the presence of diabetes (eFigure 4 in Supplement 1) or hypertension (eFigure 5 in Supplement 1).

Except in cases of mildly reduced eGFR and concomitant proteinuria, the proportion of participants who progressed to having severe CKD (eGFR <30 mL/min/1.73 m^2^) or who started dialysis therapy was relatively small (0.2% [126 participants] for severe CKD and 0.03% [85 participants] for dialysis therapy) over the 5 examined years (eFigure 6 in Supplement 1). We conducted additional analyses using a detailed classification of the CKD stage according to the KDIGO guidelines.^18^ However, due to the small sample representing advanced CKD stages (only 0.22% [178 participants] with eGFR <45 mL/min/1.73 m^2^), estimates of excess health care utilization in these groups were unstable (eFigure 7, eFigure 8, and eFigure 9 in Supplement 1). This study suggests a significant excess utilization of antihypertensive and antidiabetic drugs associated with mildly decreased eGFR and proteinuria (eFigure 10 in Supplement 1). The top 10% of health care spenders account for a significant portion of the total spending across CKD stages, as depicted in eFigure 11 in Supplement 1.

## Discussion

In this study, we examined excess health care utilization according to proteinuria and eGFR in individuals with early-stage CKD using nationwide health checkup data in Japan and found that a substantial percentage of participants (5.3%) had early-stage CKD (eGFR 30-59 mL/min/1.73 m^2^ or proteinuria) based on the screening results in the general population. Excess health care utilization owing to proteinuria and mildly reduced eGFR was consistently observed over the 5 examined years. The combination of proteinuria and mildly reduced eGFR was associated with excess health care spending and its increase over the 5 examined years. These results, demonstrating the significant association of CKD with health care utilization overspending, shed light on the importance of public health and clinical actions and interventions to prevent the occurrence and stop the progression of CKD.

Excess health care spending was less pronounced in early-stage than in advanced-stage CKD. A major strength of this study was that we could examine the impact of early-stage CKD on health care spending using data from the annual health checkup program, which had been widely conducted in Japan. Given that asymptomatic patients without diagnoses with early-stage CKD are not recorded in hospital or medical claims data, no previous analyses that we know of conducted among patients with a CKD diagnosis considered the overall burden of CKD. Our study added knowledge concerning the burden of early-stage CKD.

Mildly reduced eGFR, proteinuria, and their combination were independently associated with excess health care spending even after adjusting for hypertension and diabetes. We found consistent excess health care spending in the subgroup analyses considering the presence of hypertension and diabetes. Hypertension and diabetes are widely known diseases with a heavy social burden that increase health care utilization; however, this study revealed the need to consider CKD, which is often associated with these diseases. Additionally, excess health care spending was greater in individuals aged younger than 60 years than in those aged 60 years or older. In the older population, the decline in kidney function is associated with aging itself, so a mildly reduced eGFR may not indicate a pathological state. The clinical significance of eGFR should be considered in light of age.

Using longitudinally collected data, we could examine both the cross-sectional and longitudinal impact of CKD on health care utilization. We found that excess health care spending was constantly needed in patients with early-stage CKD compared with those without throughout the 5-year follow-up. We also found that the longitudinal increase in excess health care spending was greater with the combination of proteinuria and mildly reduced eGFR than with either one alone. The progression of CKD during the follow-up period may have influenced the increase in excess health care spending over time.

This study shows the varied aspects of excess health care utilization associated with early-stage CKD, including outpatient care, medication use, and inpatient care. CKD-related practices are complex, necessitating extensive investigation to pinpoint effective medical interventions that can help reduce excess health care utilization. For example, while pharmacological interventions such as angiotensin receptor blockers^24^ and sodium glucose cotransporter 2 inhibitors^25^ may initially lead to increased health care spending, they hold the potential to decrease long-term health care utilization. Additionally, given that CKD is a known risk factor for cardiovascular disease (CVD),^26^ the excess health care utilization may, in part, be attributed to CVD. Consequently, implementing strategies to prevent CVD may help to reduce excess health care utilization among patients with CKD. There is already a wealth of robust evidence supporting various CVD prevention strategies, such as blood pressure control, lipid management, and smoking cessation, which could be effectively tailored and implemented in this population.^27^ In addition, considering that a significant portion of health care spending is concentrated among high utilizers (eFigure 11 in Supplement 1), targeting interventions at this group may be effective.^28^

### Limitations

This study has some limitations. First, there may have been misclassification of the CKD stage because of using a single test that may have affected the observed results. Second, we were unable to adjust for access to health care; people who have poor access to health care tend to spend less on health care and might have a worse stage of CKD. However, we assessed a population whose access to health care was ensured by universal health care in Japan; thus, it is unlikely that these factors affected the results. Third, the generalizability of our results may be limited because we conducted this analysis in a predominantly male working-age Japanese population. Future studies are warranted to reexamine the impact of CKD on excess health care spending in different populations. Fourth, the analysis was adjusted for history of diabetes and hypertension, but whether these should be adjusted for should be considered carefully; they are the result of CKD progression and the cause of CKD. If we adjust for variables that are the result of CKD, the association of CKD with utilization is underestimated. We calculated excess spending that was not adjusted for hypertension and diabetes in the sensitivity analysis and found similar results. Fifth, since this study focused on early CKD stages, caution should be exercised when generalizing the results to advanced CKD stages. Sixth, we acknowledge the vital importance of identifying specific CKD-related practices that could lead to preventable excess health care utilization. Unfortunately, our data set did not provide the necessary granularity to comprehensively dissect health care spending. Seventh, the present study is observational, and thus, caution should be exercised when inferring causal relationships. Specifically, we have not demonstrated that lowering the CKD stage causally reduces excess health care utilization. Eighth, multiplicity of outcomes and the span of several years of analysis increase the likelihood of α error. As a result, we have opted to use 99% CIs for our primary analysis to account for this issue. Ninth, this study defined proteinuria using data from dipstick tests. Previous research^29^ has indicated that dipstick results of 1 or greater have a sensitivity of 98.9% and specificity of 92.6% for detecting positive proteinuria (albumin-creatinine ratio ≥300 mg/g). There is a possibility that our study may have missed cases of proteinuria, potentially leading to an underestimation of the differences in excess health care utilization among CKD stages. However, urine dipstick tests are frequently used in large-scale epidemiological studies due to their cost-effectiveness and practicality. Despite this, the findings of this study should be interpreted with caution, taking into account its limitations. Additionally, the dropout of insurance coverage due to the progression of CKD (ie, retirement) can lead to an underestimation of excess health care spending.

## Conclusions

To our knowledge, this was the first study to examine excess health care spending attributable to early-stage CKD in the general population, utilizing both a cross-sectional and a longitudinal design. Excess health care spending was observed consistently over the 5 examined years in individuals at an early stage of CKD, and there was an increasing trend throughout 5 years of follow-up in those with a combination of proteinuria and mildly reduced eGFR. We found similar results regarding excess outpatient days and excess days of hospitalization. Our findings suggest the importance of designing actions to prevent the occurrence and progression of CKD and reduce the health care burden associated with CKD for a sustainable health care system.

## Notes

Supplement 1. **eFigure 1. **Excess Healthcare Spending at Baseline for Each CKD Stage Without Adjustment for Hypertension and Diabetes **eFigure 2. **Excess Healthcare Spending at Baseline for Each CKD Stage by Age **eFigure 3. **Excess Healthcare Spending at Baseline for Each CKD Stage by Sex **eFigure 4. **Excess Healthcare Spending at Baseline for Each CKD Stage by the Presence of Diabetes **eFigure 5. **Excess Healthcare Spending at Baseline for Each CKD Stage by the Presence of Hypertension **eFigure 6. **Progression of CKD Over 5 Years by Baseline CKD Stages **eFigure 7. **Excess Healthcare Spending for More Detailed CKD Stages **eFigure 8. **Excess Days of Outpatient Care for More Detailed CKD Stages **eFigure 9. **Excess Incidence of Hospitalization for More Detailed CKD Stages **eFigure 10. **Excess Use of Antihypertensive Drugs and Antidiabetic Drugs for Early-Stage CKD **eFigure 11. **Distribution of Total Healthcare Spending Across CKD Stages

Supplement 2.
Data Sharing Statement
