# Supplementary material for: Early-Stage Chronic Kidney Disease and Related Health Care Spending
Source: JAMA Netw Open. 2024 Jan 12;7(1):e2351518. doi: 10.1001/jamanetworkopen.2023.51518 (PMC10787321; doi:10.1001/jamanetworkopen.2023.51518)
Supplement: Supplement 1. — eFigure 1. Excess Healthcare Spending at Baseline for Each CKD Stage Without Adjustment for Hypertension and Diabetes eFigure 2. Excess Healthcare Spending at Baseline for Each CKD Stage by Age eFigure 3. Excess Healthcare Spending at Baseline for Each CKD Stage by Sex eFigure 4. Excess Healthcare Spending at Baseline for Each CKD Stage by the Presence of Diabetes eFigure 5. Excess Healthcare Spending at Baseline for Each CKD Stage by the Presence of Hypertension eFigure 6. Progression of CKD Over 5 Years by Baseline CKD Stages eFigure 7. Excess Healthcare Spending for More Detailed CKD Stages eFigure 8. Excess Days of Outpatient Care for More Detailed CKD Stages eFigure 9. Excess Incidence of Hospitalization for More Detailed CKD Stages eFigure 10. Excess Use of Antihypertensive Drugs and Antidiabetic Drugs for Early-Stage CKD eFigure 11. Distribution of Total Healthcare Spending Across CKD Stages [file jamanetwopen-e2351518-s001.pdf]

Supplemental Online Content

Sakoi N, Mori Y, Tsugawa Y, Tanaka J, Fukuma S. Early-stage chronic kidney disease and related healthcare spending. *JAMA Netw Open*. 2024;7(1):e2351518. doi:10.1001/jamanetworkopen.2023.51518

eFigure 1. Excess Healthcare Spending at Baseline for Each CKD Stage Without Adjustment for Hypertension and Diabetes

eFigure 2. Excess Healthcare Spending at Baseline for Each CKD Stage by Age

eFigure 3. Excess Healthcare Spending at Baseline for Each CKD Stage by Sex

eFigure 4. Excess Healthcare Spending at Baseline for Each CKD Stage by the Presence of Diabetes

eFigure 5. Excess Healthcare Spending at Baseline for Each CKD Stage by the Presence of Hypertension

eFigure 6. Progression of CKD Over 5 Years by Baseline CKD Stages

eFigure 7. Excess Healthcare Spending for More Detailed CKD Stages

eFigure 8. Excess Days of Outpatient Care for More Detailed CKD Stages

eFigure 9. Excess Incidence of Hospitalization for More Detailed CKD Stages

eFigure 10. Excess Use of Antihypertensive Drugs and Antidiabetic Drugs for Early-Stage CKD

eFigure 11. Distribution of Total Healthcare Spending Across CKD Stages

This supplemental material has been provided by the authors to give readers additional information about their work.

© 2024 Sakoi N et al. *JAMA Network Open.*

eFigure 1. Excess Healthcare Spending at Baseline for Each CKD Stage Without Adjustment for Hypertension and Diabetes

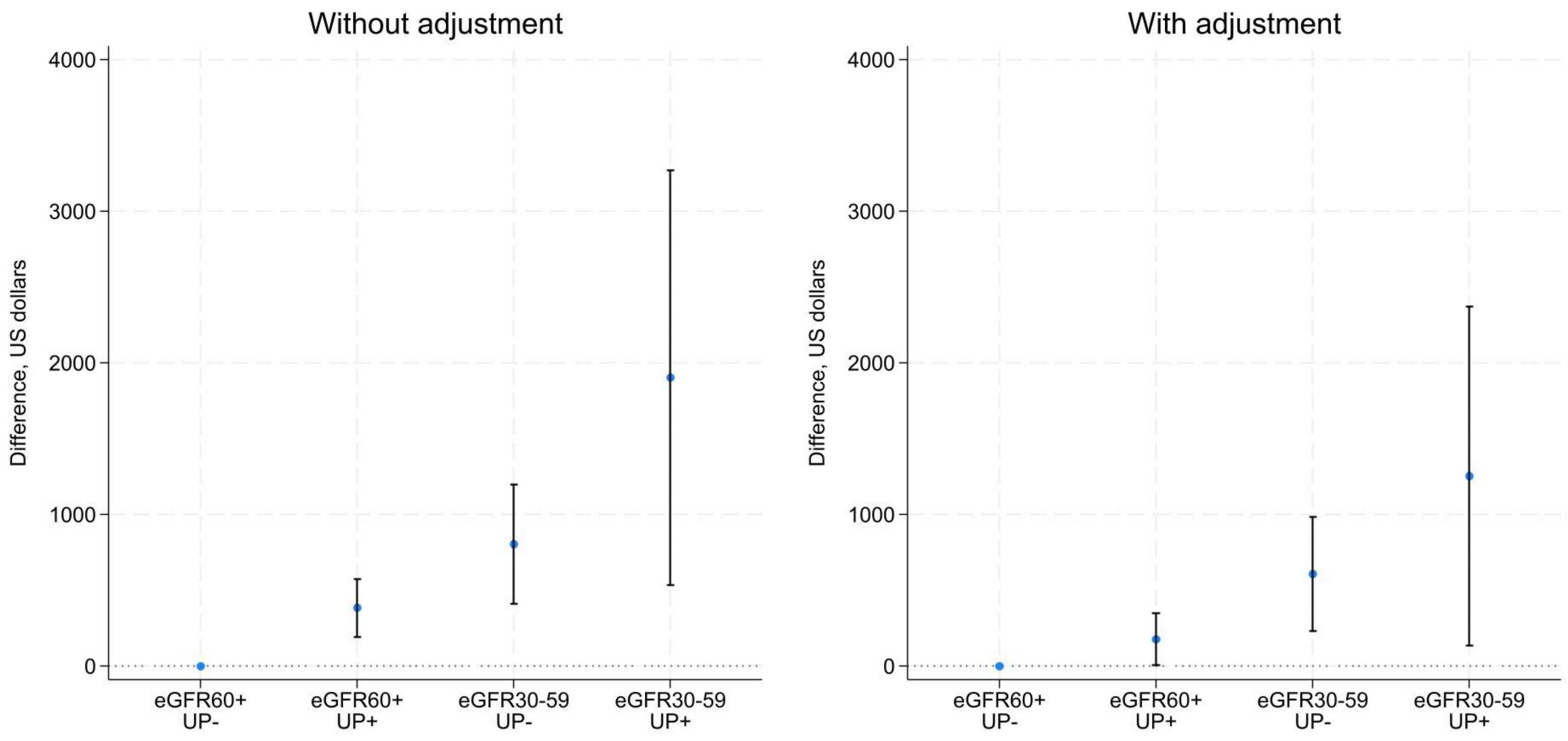

CKD: chronic kidney disease; eGFR: estimated glomerular filtration rate.

Without adjustment; adjusted only for age and sex.

With adjustment; adjusted for age, sex, hypertension, and diabetes.

Excess healthcare spending is expressed as the difference in healthcare spending (US dollars) for each stage of CKD compared to the reference group (eGFR ≥60 mL/1.73m^2 and non-proteinuria). The error bars show 99% confidence intervals.

© 2024 Sakoi N et al. *JAMA Network Open.*

eFigure 2. Excess Healthcare Spending at Baseline for Each CKD Stage by Age

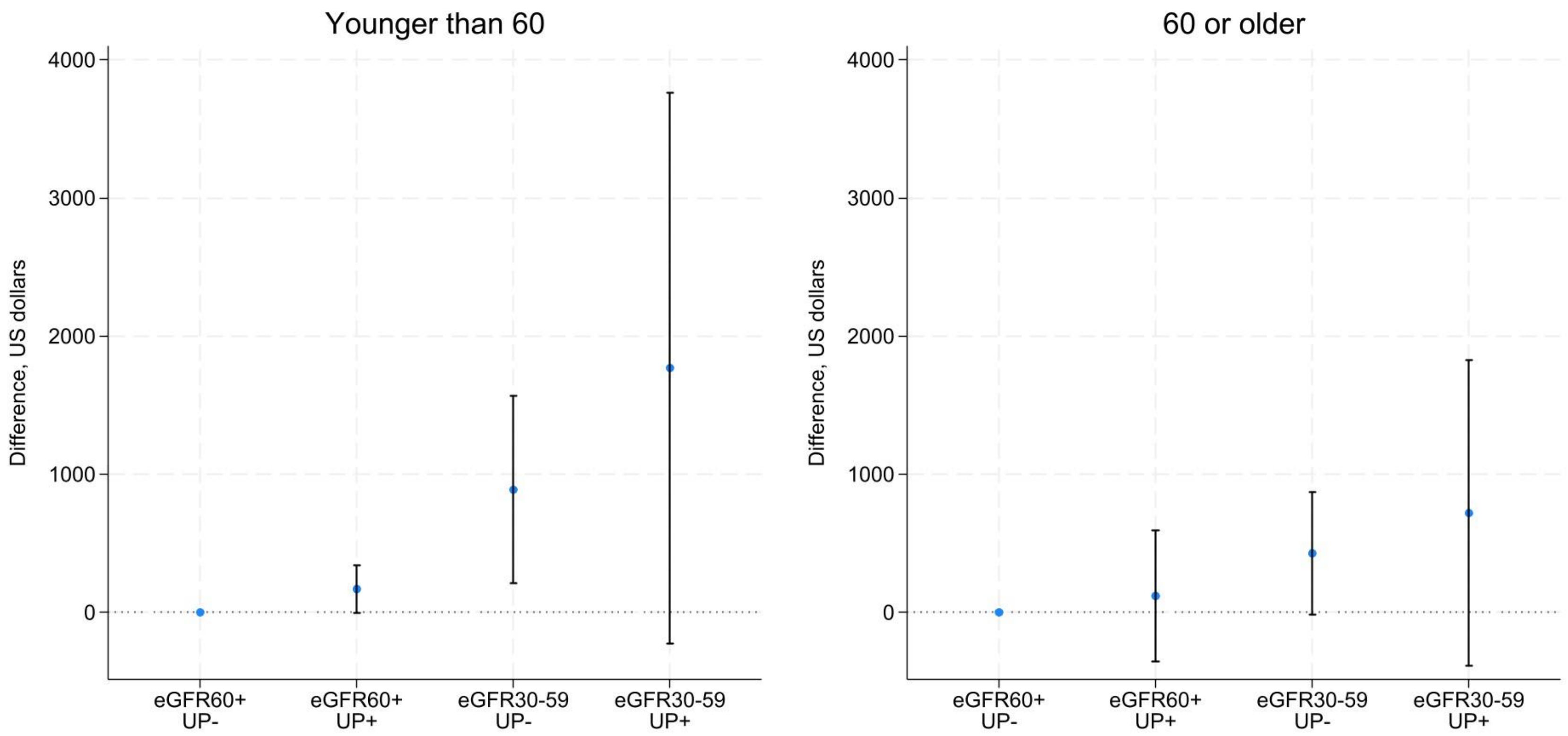

CKD: chronic kidney disease; eGFR: estimated glomerular filtration rate.

Excess healthcare spending is expressed as the difference in healthcare spending (US dollars) for each stage of CKD compared to the reference group (eGFR ≥60 mL/min/1.73m^2 and non-proteinuria), adjusted for age, sex, hypertension, and diabetes. We estimated excess healthcare spending according to the subgroup by age (younger than 60 years and 60 years or older). The error bars show 99% confidence intervals.

© 2024 Sakoi N et al. *JAMA Network Open.*

eFigure 3. Excess Healthcare Spending at Baseline for Each CKD Stage by Sex

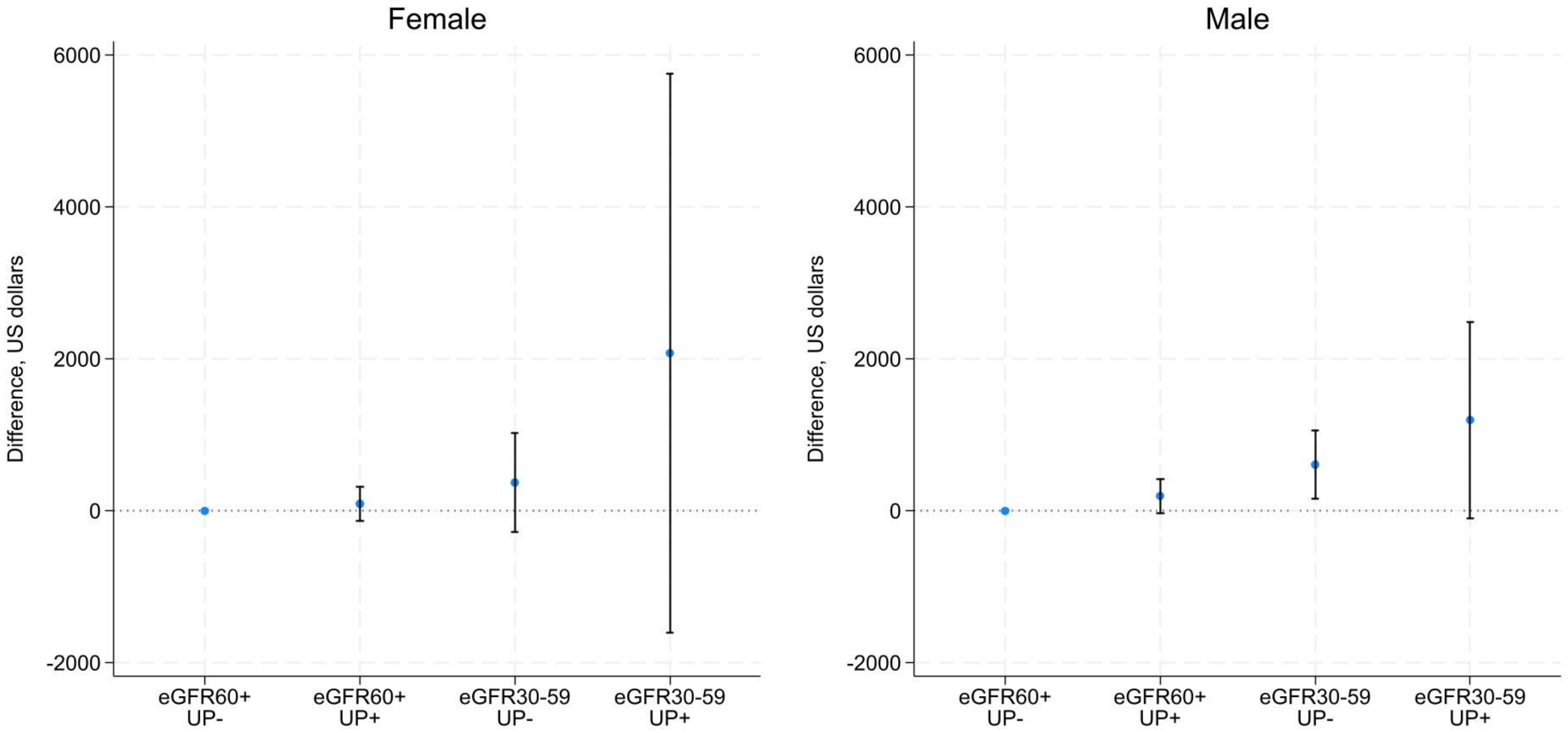

CKD: chronic kidney disease; eGFR: estimated glomerular filtration rate.

Excess healthcare spending is expressed as the difference in healthcare spending (US dollars) for each stage of CKD compared to the reference group (eGFR ≥60 mL/min/1.73m^2 and non-proteinuria), adjusted for age, hypertension, and diabetes. We estimated excess healthcare spending according to the subgroup by sex (female and male). The error bars show 99% confidence intervals.

© 2024 Sakoi N et al. *JAMA Network Open.*

eFigure 4. Excess Healthcare Spending at Baseline for Each CKD Stage by the Presence of Diabetes

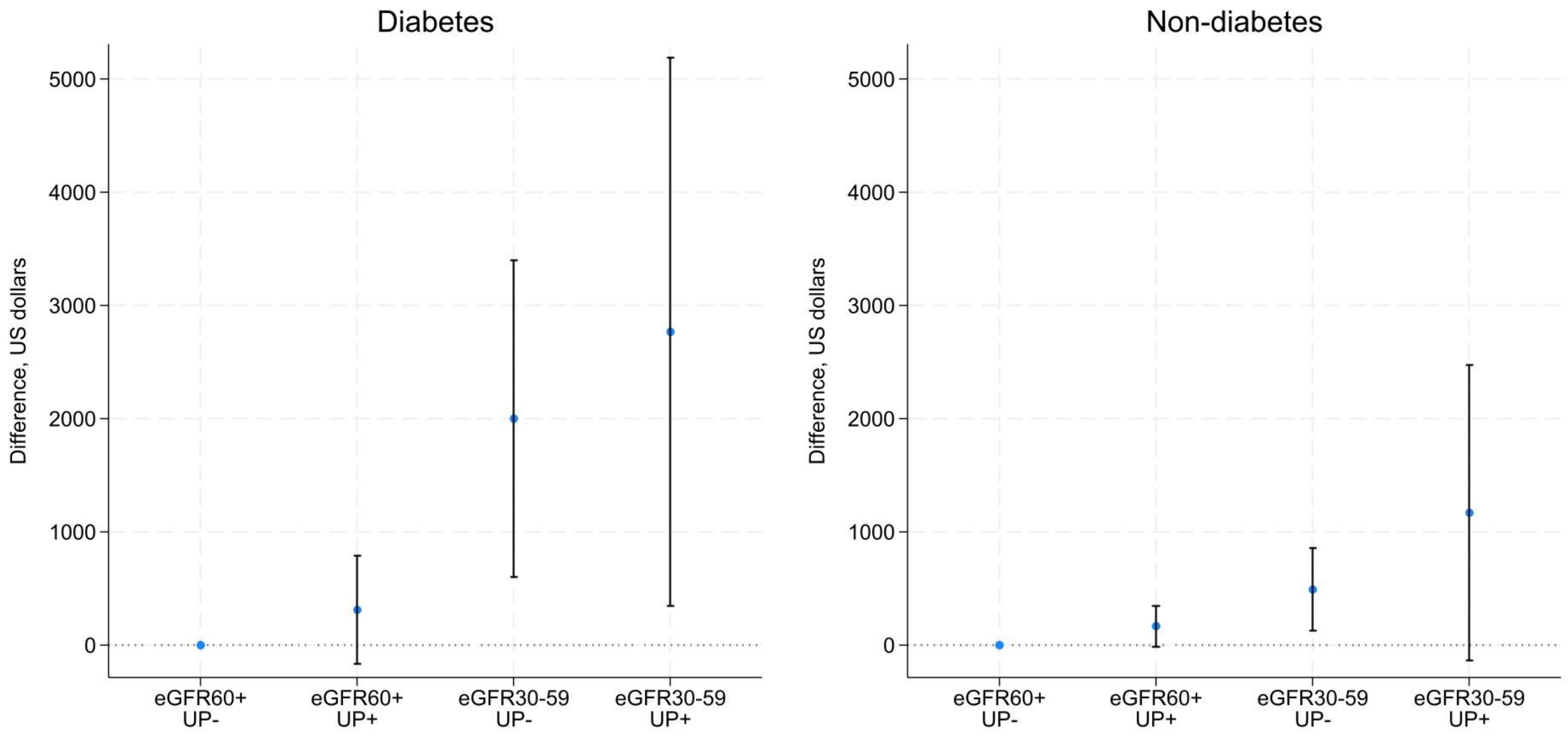

CKD: chronic kidney disease; eGFR: estimated glomerular filtration rate.

Excess healthcare spending is expressed as the difference in healthcare spending (US dollars) for each stage of CKD compared to the reference group (eGFR ≥60 mL/min/1.73m^2 and non-proteinuria), adjusted for age, sex, and hypertension. We estimated excess healthcare spending according to the subgroup by diabetes. The error bars show 99% confidence intervals.

© 2024 Sakoi N et al. *JAMA Network Open.*

eFigure 5. Excess Healthcare Spending at Baseline for Each CKD Stage by the Presence of Hypertension

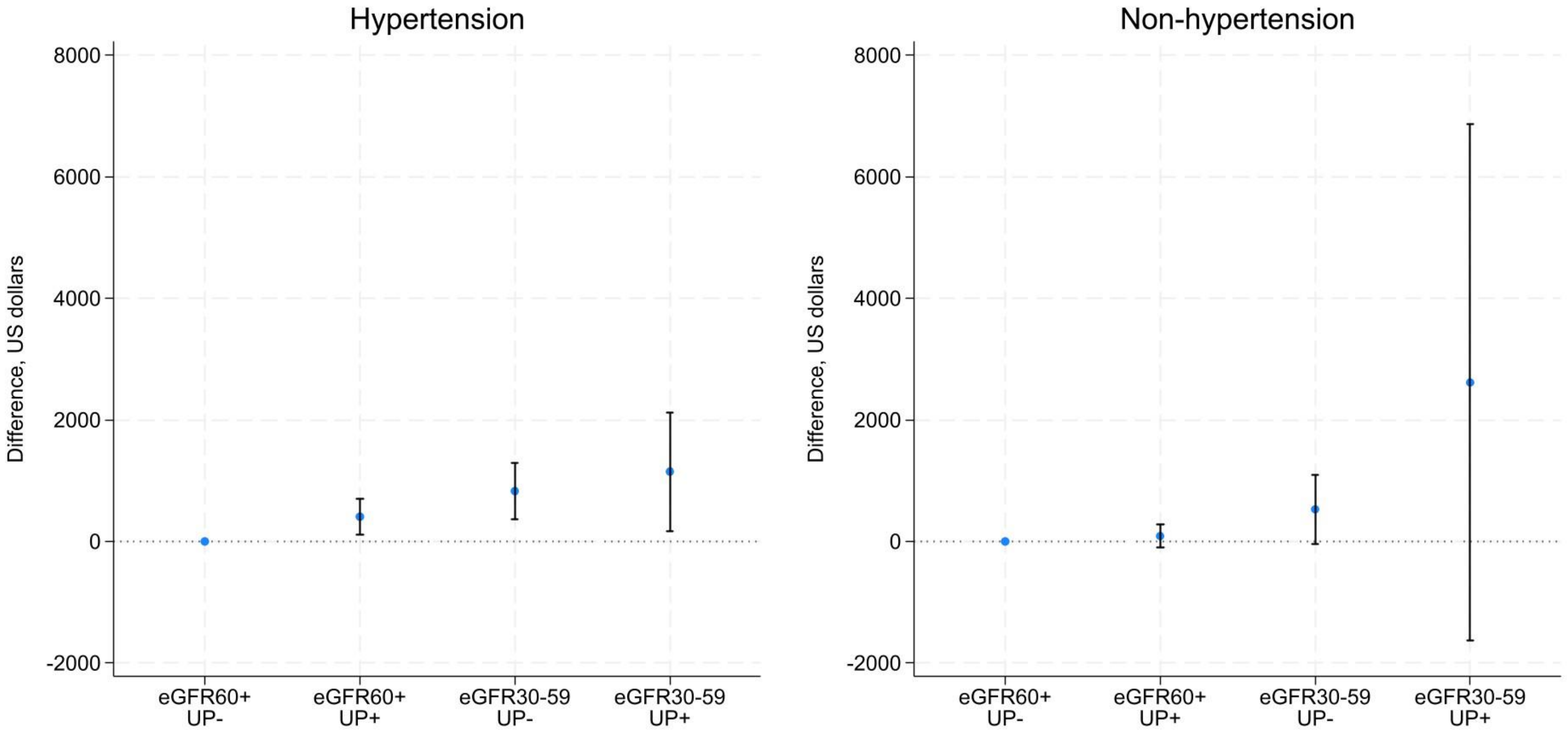

CKD: chronic kidney disease; eGFR: estimated glomerular filtration rate.

Excess healthcare spending is expressed as the difference in healthcare spending (US dollars) for each stage of CKD compared to the reference group (eGFR ≥60 mL/min/1.73m^2 and non-proteinuria), adjusted for age, sex, and diabetes. We estimated excess healthcare spending according to the subgroup by hypertension. The error bars show 99% confidence intervals.

© 2024 Sakoi N et al. *JAMA Network Open.*

eFigure 6. Progression of CKD Over 5 Years by Baseline CKD Stages

a. eGFR less than 30 mL/min/1.73m^2

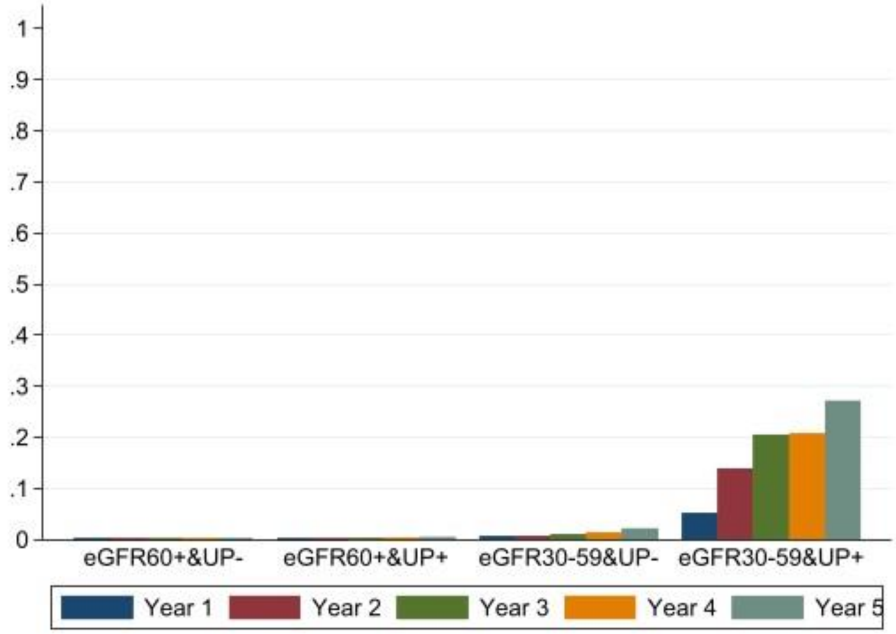

b. Started dialysis therapy.

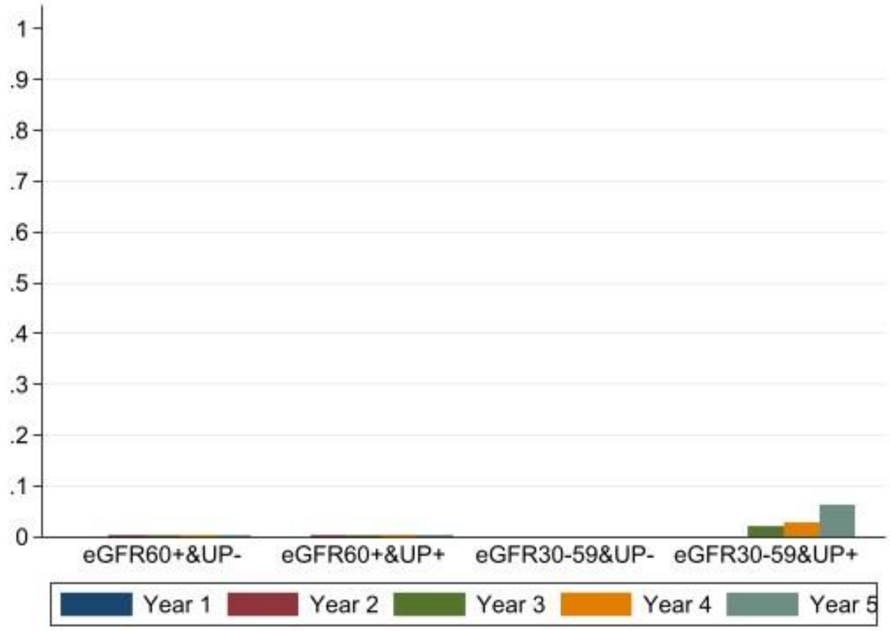

CKD: chronic kidney disease; eGFR: estimated glomerular filtration rate.

Baseline: 2014; Year 1: 2015; Year 2: 2016; Year 3: 2017; Year 4: 2018; Year 5: 2019

© 2024 Sakoi N et al. *JAMA Network Open.*

We calculated the proportion of participants who progressed to (a) an eGFR less than 30 mL/min/1.73m^2 or (b) started dialysis therapy over 5 years by baseline CKD stages.

© 2024 Sakoi N et al. *JAMA Network Open.*

eFigure 7. Excess Healthcare Spending for More Detailed CKD Stages

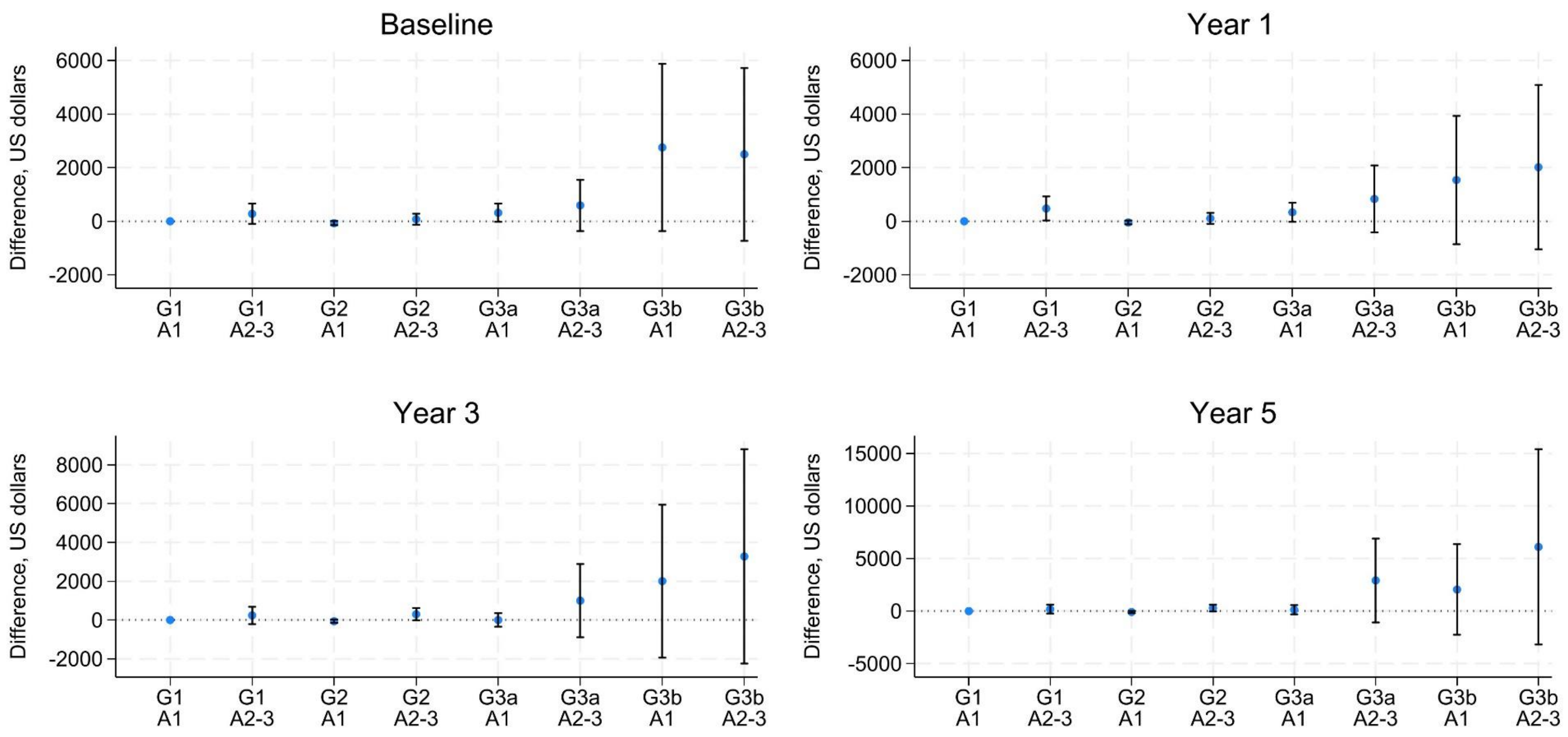

Baseline: 2014; Year 1: 2015; Year 3: 2017; Year 5: 2019

Excess healthcare spending is expressed as the difference (US dollars) in healthcare spending for each stage of CKD compared to the reference group (G1-2 & A1), adjusting for age, sex, hypertension, and diabetes.

CKD: chronic kidney disease; eGFR: estimated glomerular filtration rate; G1-2: eGFR 60 mL/min/1.73m^2 or greater; G3a: eGFR 45-59 m/min/1.73m^2; G3b: eGFR 30-44 m/min/1.73m^2; A1 non-proteinuria; A2: proteinuria 1+, A3: proteinuria 2+ or greater.

© 2024 Sakoi N et al. *JAMA Network Open.*

eFigure 8. Excess Days of Outpatient Care for More Detailed CKD Stages

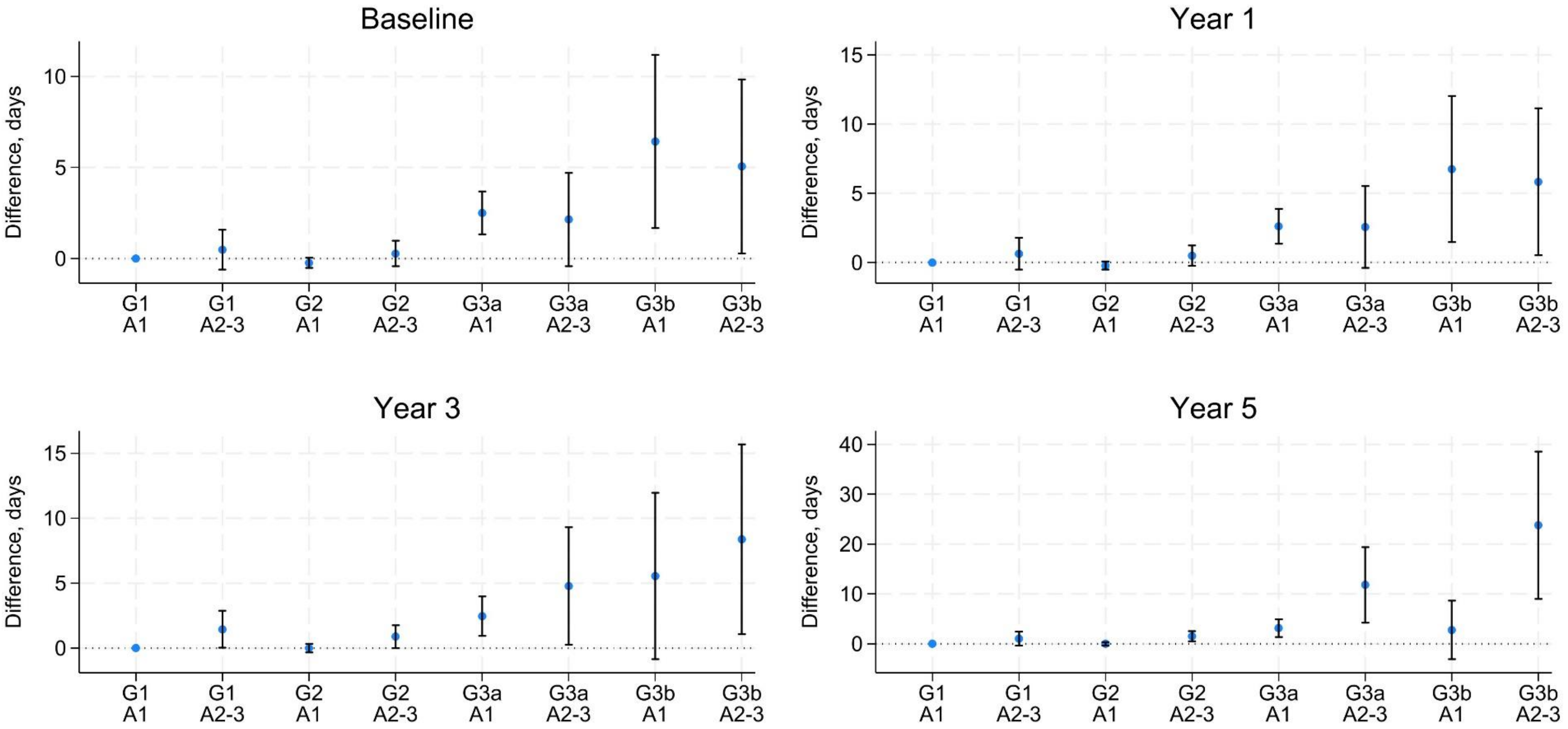

Baseline: 2014; Year 1: 2015; Year 3: 2017; Year 5: 2019

Excess days of outpatient care is expressed as the difference in the number of days of outpatient care for each stage of CKD compared to the reference group (G1-2 & A1), adjusting for age, sex, hypertension, and diabetes.

CKD: chronic kidney disease; eGFR: estimated glomerular filtration rate; G1-2: eGFR 60 mL/min/1.73m^2 or greater; G3a: eGFR 45-59 m/min/1.73m^2; G3b: eGFR 30-44 m/min/1.73m^2; A1 non-proteinuria; A2: proteinuria 1+, A3: proteinuria 2+ or greater.

© 2024 Sakoi N et al. *JAMA Network Open.*

eFigure 9. Excess Incidence of Hospitalization for More Detailed CKD Stages

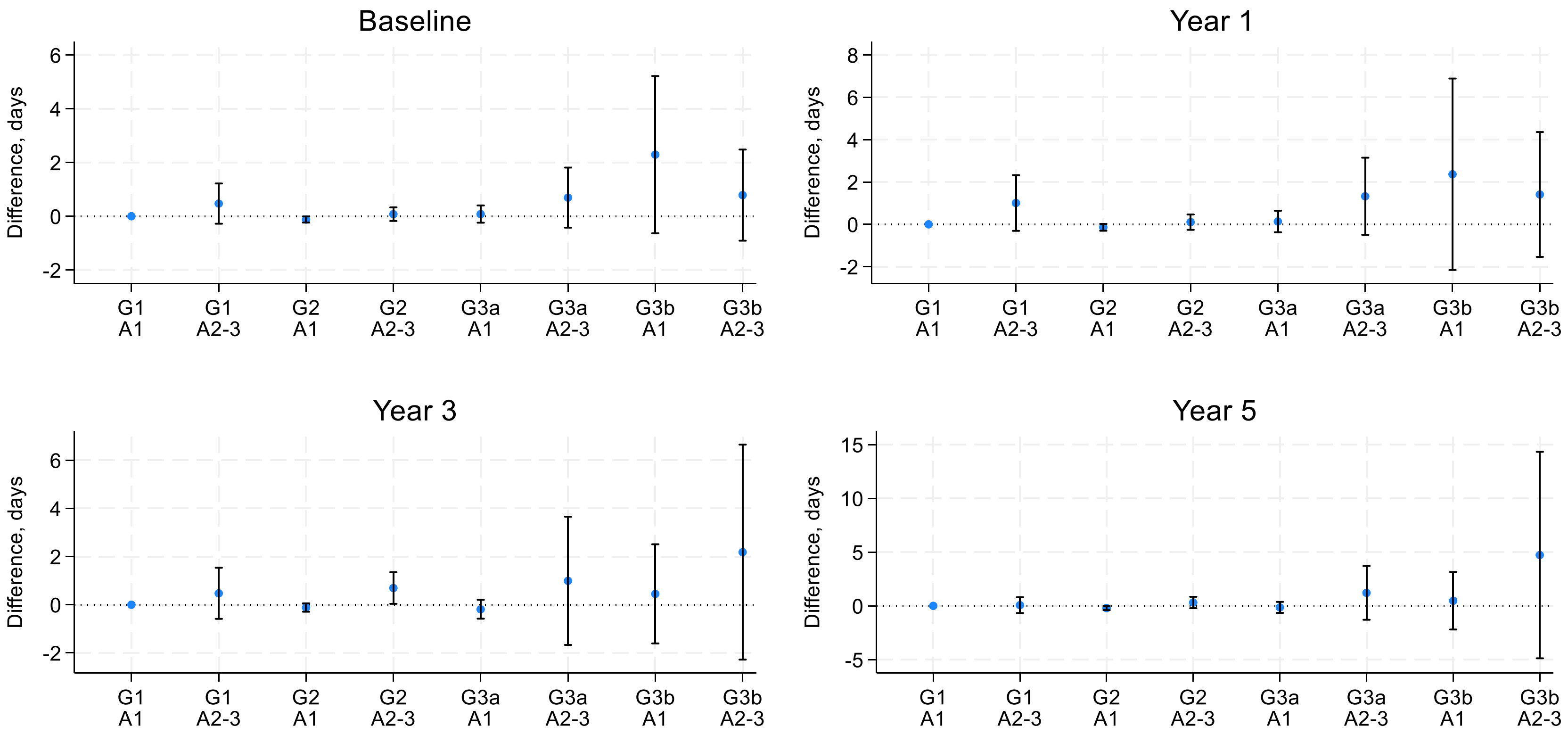

Baseline: 2014; Year 1: 2015; Year 3: 2017; Year 5: 2019

Excess incidence of hospitalization is expressed as the difference in the number of hospitalized days for each stage of CKD compared to the reference group (G1-2 & A1), adjusting for age, sex, hypertension, and diabetes.

CKD: chronic kidney disease; eGFR: estimated glomerular filtration rate; G1-2: eGFR 60 mL/min/1.73m^2 or greater; G3a: eGFR 45-59 m/min/1.73m^2; G3b: eGFR 30-44 m/min/1.73m^2; A1 non-proteinuria; A2: proteinuria 1+, A3: proteinuria 2+ or greater.

© 2024 Sakoi N et al. *JAMA Network Open.*

eFigure 10. Excess Use of Antihypertensive Drugs and Antidiabetic Drugs for Early-Stage CKD

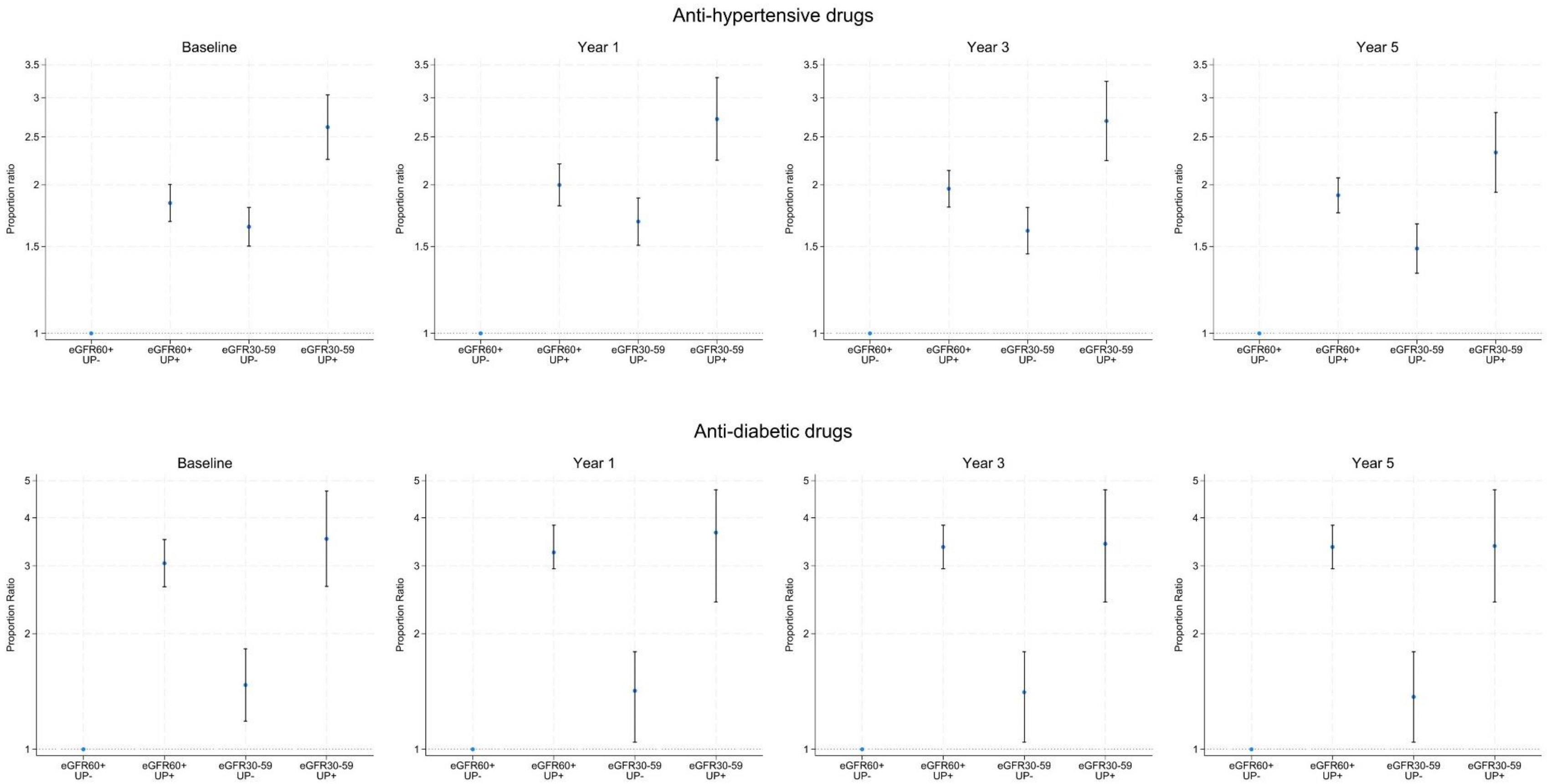

Baseline: 2014; Year 1: 2015; Year 3: 2017; Year 5: 2019

Excess use of drugs is expressed as the proportion ratio for each stage of CKD compared to the reference group (G1-2 & A1), adjusting for age, sex, hypertension, and diabetes.

CKD: chronic kidney disease; eGFR: estimated glomerular filtration rate; G1-2: eGFR 60 mL/min/1.73m^2 or greater; G3a: eGFR 45-59 m/min/1.73m^2; G3b: eGFR 30-44 m/min/1.73m^2; A1 non-proteinuria; A2-3: proteinuria 1+ or greater.

© 2024 Sakoi N et al. *JAMA Network Open.*

eFigure 11. Distribution of Total Healthcare Spending Across CKD Stages

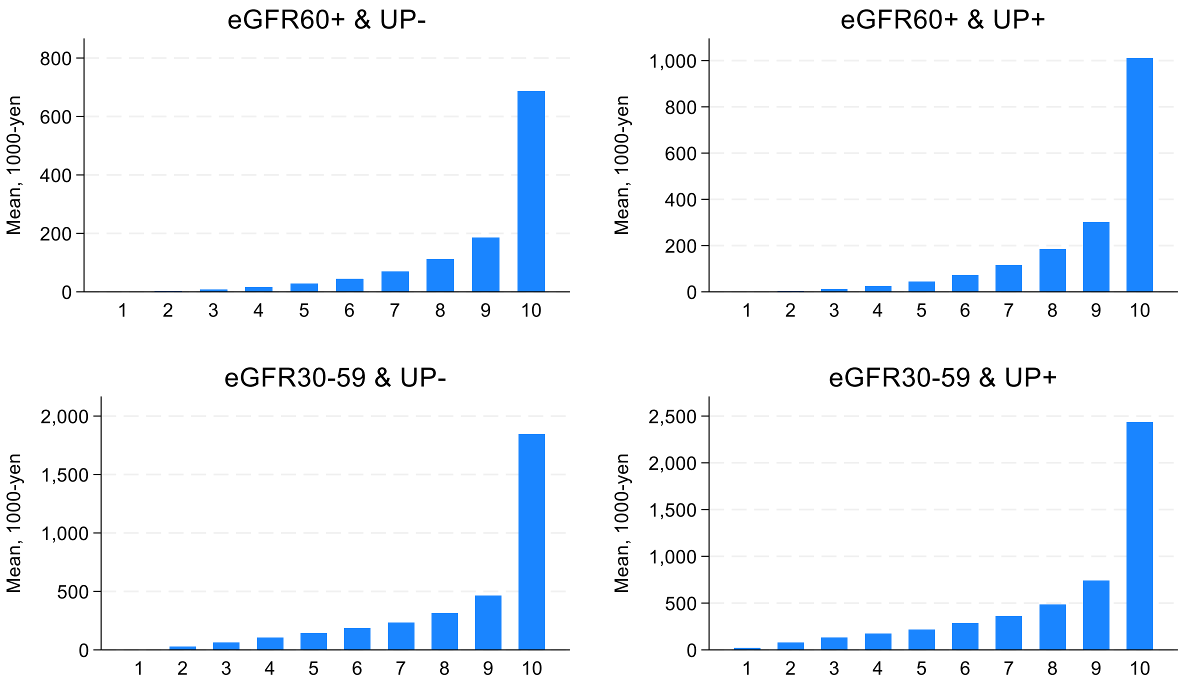

CKD stages	Median, US dollars	Interquartile range, US dollars
eGFR60+ & UP-	235	55 to 742
eGFR60+ & UP+	376	75 to 1236
eGFR30-59 & UP-	1088	428 to 2094
eGFR30-59 & UP+	1642	925 to 3190

The bar graphs show the means of total healthcare spending in each decile across CKD stages. The table shows the median and interquartile range of total healthcare spending across CKD stages. The conversion from Japanese yen to US dollars was calculated using the rate on November 11, 2023 (1 US dollar = 149.03 Japanese Yen).

© 2024 Sakoi N et al. *JAMA Network Open.*
