# Supplementary material for: Early-Stage Chronic Kidney Disease and Related Health Care Spending
Source: JAMA Netw Open. 2024 Jan 12;7(1):e2351518. doi: 10.1001/jamanetworkopen.2023.51518 (PMC10787321; doi:10.1001/jamanetworkopen.2023.51518)
Supplement: Supplement 2. — Data Sharing Statement [file jamanetwopen-e2351518-s002.pdf]

Data Sharing Statement

Sakoi. Early-Stage Chronic Kidney Disease and Related Health Care Spending. *JAMA Netw Open*. Published January 12, 2024. doi:10.1001/jamanetworkopen.2023.51518

Data
Data available: No

Additional Information
Explanation for why data not available: The data underlying this article is not shared due to the privacy policy of data providers.
